# Age‐specific spectrum of etiological pathogens for viral diarrhea among children in twelve consecutive winter‐spring seasons (2009–2021) in China

**DOI:** 10.1002/jmv.27790

**Published:** 2022-04-26

**Authors:** Gang Wang, Rui‐Qiu Zhao, Xiang Tang, Luo Ren, Yun‐Fa Zhang, Heng Ding, Yue Li, Yu‐Na Wang, Shuang Li, Ling Yu Zhang, En‐Mei Liu, Hong‐Mei Xu, Xiao‐Ai Zhang, Wei Liu

**Affiliations:** ^1^ State Key Laboratory of Pathogen and Biosecurity Beijing Institute of Microbiology and Epidemiology Beijing China; ^2^ Chongqing Key Laboratory of Pediatrics Children's Hospital of Chongqing Medical University Chongqing China; ^3^ School of Public Health Peking University Beijing China

**Keywords:** Age, China, Diarrhea, Enteric viruses, Rotavirus

## Abstract

Viral diarrhea is one of the leading causes of morbidity and mortality in children. This study was conducted to disclose the etiological cause and epidemiological features of viral diarrhea among children in China. From 2009 to 2021, active surveillance was performed on pediatric patients with acute diarrhea and tested for five enteric viruses. Positive detection was determined in 65.56% (3325/5072) patients and an age‐specific infection pattern was observed. A significantly higher positive rate was observed in 12–23‐month‐old children for rotavirus (47.46%) and adenovirus (7.06%), while a significantly higher positive rate was observed for norovirus (37.62%) in 6–11‐month‐old patients, and for astrovirus (11.60%) and sapovirus (10.79%) in 24–47‐month‐old patients. A higher positive rate of rotavirus in girls and norovirus in boys was observed only among 6–11 months of patients. We also observed more norovirus among patients from rural areas in the 0–5‐ and 36–47‐month groups and more rotavirus among those from rural areas in the 12–23‐month group. Diarrhea severity was greater for rotavirus in the 6–23‐month group and norovirus in the 6–11‐month group. Coinfections were observed in 29.26% (973/3325) of positive patients, and were most frequently observed between rotavirus and others (89.31%). Our findings could help the prediction, prevention, and potential therapeutic approaches to viral diarrhea in children.

## 1 INTRODUCTION

Globally, acute diarrhea infection remains the most frequent childhood illness and cause of attendance at health facilities, particularly in low‐income and middle‐income countries. An estimated 1 in 10 childhood deaths results from diarrhea disease during the first five years of life.^1^ Despite significant progress in reducing diarrhea mortality through vaccination intervention, improving sanitation water supply, and public health awareness, it remains 1 of the top 5 causes of death among children younger than five years.^2^

Although intensive data about the etiology and epidemiology of infectious diarrhea in children are available, the etiology differs among age groups depending on geography, climate, and economic development.^3^ However, most previous studies lacked continuous active surveillance and without comprehensive laboratory detection of enteric viruses. Particularly, limited data are available regarding the etiology of infectious diarrhea disentangled by age and other demography. An enhanced acquirement of these refined data in children would be valuable for planning and adopting targeted preventive measures. Here we conducted a long persistent active surveillance study in Chongqing, a mountainous region with dense populations and high humidity in southwestern China, to identify the age‐specific etiological, epidemiological, and clinical features of children with acute diarrhea.

## 2 MATERIALS AND METHODS

### 2.1 Patients recruitment and specimen collection

From July 2009 to April 2021, ongoing surveillance of acute diarrhea for pediatric patients of <5 years old was conducted at the Children's Hospital of Chongqing Medical University. The hospital is the largest children's hospital in southwestern China, with >2000 beds and an annual outpatient capacity of approximately 3 million people, serving patients with a wide geographic range across central and western China. During the study period, a standard guideline was administered for the patients recruited and laboratory tests. Briefly, acute infectious diarrhea was defined as three or more loose or watery stools per day and duration <2 weeks. Patients who had confirmed inflammatory bowel disease, celiac disease, cystic fibrosis, food intolerance, or patients who had any apparent clinical respiratory signs or symptoms were excluded. Disease outcome was defined as nonsevere (mild/moderate) or severe diarrhea based on the predefined criteria.^4^ Briefly, severe cases were defined as scores ≥11, and nonsevere cases were defined as scores of 0–10 according to the modified Vesikari scale. The stool specimens were collected immediately from the patients after their visiting the hospital and then stored at −80°C for a later process. The patients' information was retrieved from medical records, including demographic data, underlying medical conditions, clinical symptoms, and signs. This study was performed with the approval of the Ethics Review Committee of the Children's Hospital of Chongqing Medical University. The methods were carried out in accordance with the approved guidelines. Written informed consent was obtained from all guardians of participants.

### 2.2 Laboratory detection of stool samples

Viral DNA/RNA of six viruses (types) was tested for each of the collected samples. Briefly, stool samples were suspended in phosphate‐buffered saline, vortexed vigorously, and centrifuged at 12 000 rpm for 1 min in a microfuge. Total nucleic acids (DNA and RNA) were extracted from 140 μl of a 10% fecal supernatant using QIAamp Viral RNA Mini Kit (QIAGEN) and eluted in 20 μl DEPC water according to the manufacturer's instructions. The complementary DNA was synthesized by using the SuperScript®III First‐Strand Synthesis System for reverse transcription‐polymerase chain reaction (RT‐PCR) (Invitrogen). The real‐time RT PCR was applied to test norovirus (GI/GII) by using specific probes and primers described previously^5^ (Table S1). The RT‐PCR and PCR were applied to test astrovirus, sapovirus, and adenovirus as described previously,^6^, ^7^, ^8^ while rotavirus A was detected by using an IDEIA rotavirus direct antigen detection kit (IDEIA, Oxford, UK).

### 2.3 Statistical analysis

Continuous variables were expressed as medians and interquartile ranges (IQR) and categorical variables were expressed as numbers (%). The viral positive rate was calculated by dividing the number of at least one positive pathogen by the number of patients tested for all five viruses. Six age groups were defined: 0–5, 6–11, 12–23, 24–35, 36–47, and 48–59 months, and the positive rate among the age groups was compared by the Cochran‐Armitage trend test. Pearson chi‐square test or Fisher exact test was performed to compare other categorical variables. To infer viral interactions between any of the two tested viruses, we applied multivariable binary logistic regression models after adjusting age, sex, the time from onset to hospital admission (delay), and the monthly background prevalence.^9^ The odds ratio and its 95% confidence interval were estimated using the maximum likelihood method. A *p* value <0.05 was considered statistically significant. All statistical analysis was performed using R version 4.1.0.

## 3 RESULTS

### 3.1 Characteristics of the study population

From July 2009 to April 2021, 5330 cases of younger than five years with acute diarrhea were recruited for the current research. After excluding 258 patients with incomplete data collection or inadequate sampling, the remaining 5072 patients were tested for all five viruses. Of whom 60.25% were boys, with a median age of 10 (IQR: 6‒15) months, and the largest proportion (89.41%) was <24 months. More patients were recruited from the outpatient department (86.67%, 4396/5072) than from the inpatient department. More patients resided in urban areas (88.39%, 4483/5072) than in rural areas (Table 1).

**Table 1:** Demographic and clinical characteristics of patients with acute diarrhea in Chongqing, 2009‒2021

Characteristics	All virus tested	Any virus positive	Coinfection
	(*n* = 5072)	(*n* = 3325)	(*n* = 973)
Sex			
Boy	3056 (60.25)	2001 (60.18)	587 (60.33)
Girl	2016 (39.75)	1324 (39.82)	386 (39.67)
Age group (month)			
0–5	1036 (20.43)	525 (15.79)	147 (15.11)
6–11	1927 (37.99)	1257 (37.81)	371 (38.13)
12–23	1572 (30.99)	1198 (36.03)	359 (36.90)
24–35	319 (6.29)	224 (6.74)	67 (6.89)
36–47	139 (2.74)	83 (2.50)	24 (2.47)
36–59	79 (1.56)	38 (1.14)	5 (0.51)
Case type			
Outpatients	4396 (86.67)	2788 (83.85)	808 (83.04)
Inpatients	38 (0.75)	22 (0.66)	12 (1.23)
Unknown	638 (12.58)	515 (15.49)	153 (15.72)
Residence			
Urban	4483 (88.39)	2927 (88.03)	864 (88.80)
Rural	552 (10.88)	380 (11.43)	107 (11.00)
Unknown	37 (0.73)	18 (0.54)	2 (0.21)
Clinical characteristics			
Vomiting	2276 (44.87)	1821 (54.77)	539 (55.40)
Fever	984 (19.40)	652 (19.61)	178 (18.29)
Temperature	38.5 (38.0–39.0)	38.5 (38.0–39.0)	38.5 (38.0–39.0)
Duration of diarrhea (days)^a^	3.0 (2.0–5.0)	3.0 (2.0–4.0)	3.0 (2.0–4.0)
Frequency of diarrhea (times)^a^	5.0 (3.5–6.5)	4.5 (3.5–6.5)	5.0 (3.5–6.5)
Duration of vomiting (days)^a^	1.0 (1.0–2.0)	1.0 (1.0–2.0)	1.0 (1.0–2.0)
Frequency of vomiting (times)^a^	3.0 (2.0–4.0)	3.0 (2.0–4.0)	3.0 (2.0–4.0)
Stool character			
Watery	3399 (67.01)	2229 (67.04)	597 (61.36)
Mushy	249 (4.91)	129 (3.88)	39 (4.01)
Mucus	44 (0.87)	21 (0.63)	5 (0.51)
Bloody	10 (0.20)	3 (0.09)	1 (0.10)
Unknown	1370 (27.01)	943 (28.36)	331 (34.02)
Severity			
Nonsevere	4342 (85.61)	2764 (83.13)	806 (82.84)
Severe	730 (14.39)	561 (16.87)	167 (17.16)
Delay, median^a^	3.0 (1.0‐5.0)	3.0 (1.0‐4.0)	3.0 (1.0‐4.0)

*Note*: Data are *n* (%) unless otherwise indicated.

Abbreviation: IQR, interquartile range.

^a^ Data are median (IQR).

The most common symptom that accompanied diarrhea included vomiting and fever ≥37.5°C, which was reported in 44.87% (2276/5072) and 19.40% (984/5072) of the patients at the time of physical examination. Of the 5072 tested stool specimens, watery stool (67.01%) was the most commonly seen, followed by mushy stool (4.91%), mucus stool (0.87%), and bloody stool (0.20%). The median duration from onset to first hospital admission was 3 days (IQR: 1‒5). Severe disease outcome was developed in 14.39% (730/5072) of the patients (Table 1).

### 3.2 The age‐specific pattern of viral infection

**Figure 1:**
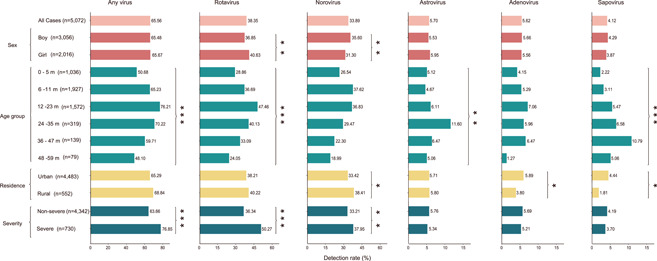
The enteric virus detection rate in pediatric patients with acute diarrhea. The lengths of colored bars indicate the detection rate of each pathogen by sex, age group, residence, and severity. The same filled colors of bars mean they are in the same group being compared. The number next to the group name shows the number of cases tested. The Chi‐square test or Fisher's exact test was used for comparison between groups by sex, residence and severity, and the Cochran‐Armitage trend test was used for comparison among age groups (*p < 0.05; **p < 0.01; ***p < 0.001)

Overall, 65.56% (3325/5072) patients were positive for at least one enteric virus, and viral coinfection was determined in 973 (19.18%) cases. The most prevalent virus was rotavirus (38.35%, 1945/5072), followed by norovirus (33.89%, 1719/5072), astrovirus (5.70%, 289/5072), adenovirus (5.62%, 285/5072), and sapovirus (4.12%, 209/5072) (Figure 1 and Table S2–S3). All viruses except for astrovirus displayed decreased detection as the interval from disease onset to visit increased (Figure S1).

All the enteric viruses were determined in all age groups, however, their infection patterns differed regarding age. When all age groups were counted, the positive rate increased rapidly with age, peaking at age 12–23 months and then decreased (Cochran‐Armitage trend test, *p* < 0.001). A significantly higher viral positive rate was observed in 12–23 months old children (76.21%, 1198/1572) than in other age groups, especially with a higher rate of rotavirus (47.46%, 746/1572) and adenovirus (7.06%, 111/1572). In contrast, a significantly higher positive rate for norovirus was observed in 6–11 months old children (37.62%, 725/1927), while for astrovirus (11.60%, *n* = 37/319) and sapovirus (10.79%, *n* = 15/139) was observed in 24–47 months old children (Figure 1 and Table S3).

Although the overall viral infection was comparable between boys and girls (65.48% vs. 65.67%, *p*  =  0.885), gender discrepancy was observed in specific age groups. For example, for the 6–11 months group, the prevalence of rotavirus was higher in girls than in boys (39.47% vs. 34.90%, *p* = 0.042), while the norovirus was higher in boys (39.76% vs. 34.30%, *p* = 0.016). This difference was not observed for other enteric viruses (all *p* > 0.05) (Figure 1 and Table S3–S4).

The positive rates of viral infections in patients living in urban and rural areas were 65.29% (2927 of 4483) and 68.84% (380 of 552), respectively, displaying no significant difference (*p*  =  0.097, Table S3). When the geographic difference was further compared regarding age, we observed a significantly higher prevalence of norovirus among patients living in rural areas in 0–5 months (34.92% vs. 25.36%, *p* = 0.023) and 36–47 months groups (71.43% vs. 19.85%, *p* = 0.007), while rotavirus was more frequently identified among those living in rural areas only in 12–23 months group (55.15% vs. 46.74%, *p* = 0.041). No such geographic difference was observed for the other enteric viruses across all age groups (Table S5).

Severe patients had significantly higher overall rate of viral infection than nonsevere patients (76.85% vs. 63.66%, *p* < 0.001), also higher rate for rotavirus (50.27% vs. 36.34%, *p* < 0.001) and norovirus (37.95% vs. 33.21%, *p* = 0.012) (Table S3). When the age‐specific rate was further disaggregated, we only observed a higher rate of rotavirus in the 6–11 months and 12–23 months groups, and a higher rate of norovirus in the 6–11 months group (Table S6).

### 3.3 Temporal/seasonal trends of viral etiologies

**Figure 2:**
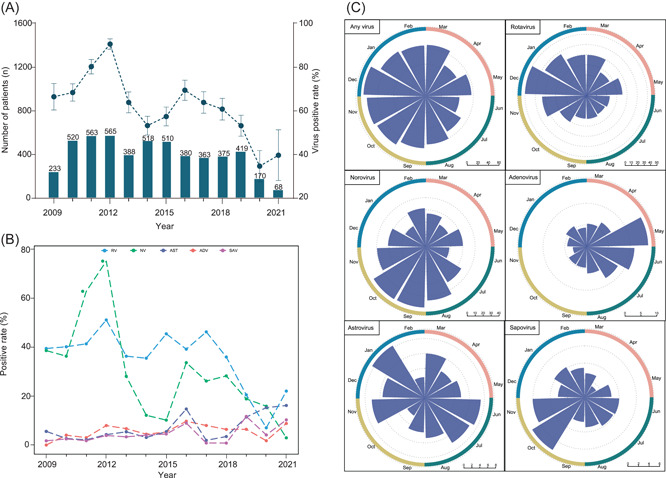
Annual trends and seasonal distribution characteristics of five enteric viruses in pediatric patients with acute diarrhea. (A) The annual number of patients with acute diarrhea and the positive rate of any virus‐positive. The positive rate and its 95% confidence interval of viral pathogens each year were indicated. The positive rate was calculated by dividing the number of at least one positive pathogen by the number of patients tested for all five viruses. (B) The annual trend of each of the five viruses of acute diarrhea in children. (C) Seasonal prevalence characteristics of five specific viruses with acute diarrhea in children. The radius of the arc indicates the positive rate of each virus. The red ring represents spring, the dark green ring represents summer, the yellow ring represents autumn, and the blue ring represents winter

During the study period from 2009 to 2021, the overall positive rate had fluctuated across the study years, showing an upward trend from 2009, attaining the peaking level in 2012 and followed thereafter by decreased rate, to the lowest level in 2020 when the COVID‐19 epidemic was noticed (Figure 2A). A similar dynamic trend was observed for rotavirus and norovirus, with both peaks of infection observed in 2012 (Figure 2B). No clear temporal trends for infection of the other viruses, which was highly likely due to low detection rates (Figure 2B).

An obvious seasonal pattern was observed for the overall positive rate, with a higher level of circulation observed in autumn (70.51%, 1246/1767) and winter (70.30%, 1257/1788) than in spring (55.97%, 431/770) and summer (52.34%, 391/747) (*p* < 0.001, Figure 2C and Table S7). A similar seasonal pattern was observed when each of the tested viruses was separately analyzed, with all but adenovirus showed a higher circulation level during either winter or autumn or during both seasons.

### 3.4 Viral coinfection and interaction patterns

**Figure 3:**
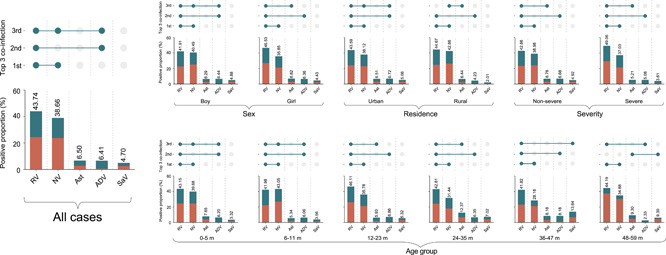
The coinfection patterns of enteric viruses in pediatric patients with acute diarrhea. The proportion of each positive pathogen was noted in % and by the length of colored bars. The orange bar indicates viral mono‐infection; the green bar indicates coinfection. The three most common coinfections were presented above the constituent ratios

Viral coinfection was detected in 973 cases, accounting for 29.26% of the total 3325 positive patients. Of whom 832 had dual‐infection, 133 had triple‐infection, and 8 had more than four enteric viruses. The highest coinfection rate was seen among the age group of 12–23 months (22.84%), followed by the 24–35 months group (21.00%), 6–11 months (19.25%), and 36–47 months (17.27%) (Figure S2A). It's not surprising that the top‐ranking coinfection occurred between rotavirus‐norovirus, the two most frequently determined viruses, which remained the same across all age groups (Figure 3 and Figure S2B). The secondly ranking coinfection in most age groups occurred between rotavirus and adenovirus except for 48–59 months old patients. The third coinfection differed among age groups: rotavirus‐norovirus‐adenovirus in 0–11 months, rotavirus‐astrovirus in 12–35 months, rotavirus‐sapovirus in 36–47 months, and rotavirus‐adenovirus in 48–59 months (Figure 3).

**Figure 4:**
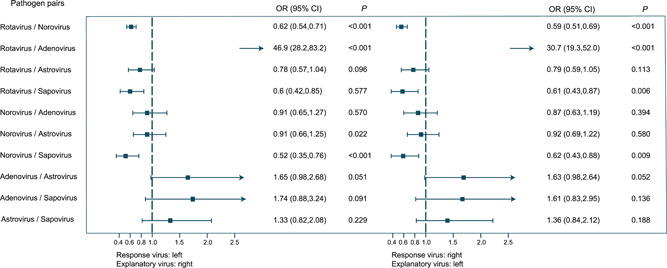
The interaction patterns of enteric viruses in pediatric patients with acute diarrhea. The box indicates the estimated odds ratio and the interval represents the 95% confidence interval of the odds ratio. Odds ratio estimates for ten pathogen‐pair interactions were generated from five virus logistic regression models by adjusting for the effects of age, sex, delay, and the monthly background prevalence of response virus infections. The left forest plot selected the virus to the left of the pathogen pair as the response virus, and the right forest plot did the opposite

The viral interaction pattern was determined by logistic regressions, which revealed potential synergistic/competitive interactions among the tested viruses. Most of the interactions were negative, occurring between rotavirus‐norovirus, rotavirus‐sapovirus, norovirus‐sapovirus. Positive interaction was only seen between rotavirus‐adenovirus (Figure 4).

## 4 DISCUSSION

In this study, by performing a systematic, continuous, laboratory‐based surveillance of acute viral diarrhea on 5072 children in Chongqing, we disclosed an age‐specific infection pattern of five dominant enteric viruses among those aged 0–59 months, with the highest prevalence of viral infection occurring at 12–23 months. The finding is consistent with previous studies indicating high susceptibility to viral infection in early childhood, especially at the age younger than five years.^10^, ^11^

Consistent with previous findings in China^10^, ^11^, ^12^, ^13^ as well as other countries, e.g., in northeast Brazil before the introduction of rotavirus vaccination,^14^, ^15^ rotavirus remained the top viral pathogen, and norovirus was next ranked. It's been well accepted that most children might develop three or more episodes of rotavirus infections by age of two years, and thereafter when host immunity has been established, a steadily decreasing infection occurs in the older childhood.^16^ By applying an age‐specific analysis, we determined norovirus had replaced rotavirus to become the top etiological cause of acute diarrhea in children aged 6–11 months, despite a slight excess over rotavirus. This might as well indicate an increased incidence of norovirus among this age group owing to the increased transmission in the household, such as immunocompromised elderly who are more likely to live in the same house as the children in a traditional Chinese family.

The ranking of other viruses differed among age groups. Children aged 24–47 months were the most susceptible group to astrovirus and sapovirus infection, while adenovirus infections were most frequently determined in 12–23 months. The discrepancy among age groups was primarily related to host immunity. As shown in previous studies, the first infection of these viruses provided moderate protection against subsequent infections.^17^, ^18^ Still, the lower prevalence made them less likely to be exposed to children, so the protective immunity was developed later than rotavirus.

Previous studies in China have indicated the unique characteristics of some specific enteric viruses associated with acute diarrhea in rural areas.^19^, ^20^ However, age‐specific differences in the features of the enteric viruses of acute diarrhea between urban and rural areas have not been well demonstrated, especially in children. In our study, the comparison between rural and urban patients disclosed age‐specific differences in the viral pathogen spectrum in which the positive rates of rotavirus in the 12–23 months group and norovirus in the 0–5 months and 36–47 months groups were higher in rural patients than in urban patients. Although hygiene of food/water supply was improved in recent years, rotavirus and norovirus still need more attention in routine clinical diagnosis and vaccination programs in rural areas.

In our study, viral coinfection accounted for 29.26% of the total positive detection. Rotavirus‐norovirus was the most commonly determined coinfection, followed by rotavirus‐adenovirus and rotavirus‐norovirus‐adenovirus. We also observed a significant positive interaction between rotavirus and adenovirus using the logistic regression model after adjusting age, sex, delay, and the monthly background prevalence. This finding could be supported by previous studies, albeit the reason remained obscure.^21^, ^22^ The rest of the interactions were negative, including rotavirus‐norovirus, rotavirus‐sapovirus, norovirus‐sapovirus. The possible mechanisms were disclosed by previous epidemiological and mechanism studies. Nguyen et al. demonstrated that the binding with histo‐blood group antigens plays a critical role in both norovirus and rotavirus infections in children,^23^ possibly resulting in the competitive interaction. Another study suggested that rotavirus infection is susceptible to interference by other viral pathogens in the gut, resulting in reduced virus replication.^24^

Based on active surveillance performed on pediatric patients with acute diarrhea in 12 consecutive winter‐spring seasons, an age‐specific infection pattern of five dominant enteric viruses was displayed for the 0–59‐month‐old pediatric patients. This comprehensive data might assist in the planning of an integrated control program for the leading causes of acute viral diarrhea and the children that are susceptible to the predominant enteric viruses. It might also provide evidence for informing policymakers on the future vaccine and intervention development in this area. The current study also emphasizes the need for ongoing comprehensive surveillance of vulnerable children's groups. A more precise investigation in deciphering the prevalence among age, sex, and regions is warranted in the future.

## AUTHOR CONTRIBUTION

**Wei Liu, Xiao‐Ai Zhang, and Hong‐Mei Xu:** conceived and designed the study. **Rui‐Qiu Zhao, Xiang Tang, Luo Ren, En‐Mei Liu, Hong‐Mei Xu, Wei Liu, and Xiao‐Ai Zhang:** collected samples and data. **Gang Wang, Rui‐Qiu Zhao, Yun‐Fa Zhang, Heng Ding, Yue Li, Yu‐Na Wang, Ling Yu Zhang, and Shuang Li:** performed the experiments. **Wei Liu, Xiao‐Ai Zhang, and Gang Wang:** analyzed the data. **Wei Liu, Xiao‐Ai Zhang, Hong‐Mei Xu, Gang Wang, and Rui‐Qiu Zhao:** drafted the manuscript. All authors read and approved the final paper.

## CONFLICTS OF INTERESTS

The authors declare no conflict of interest.

## Supporting information

Supporting information.
