# Supplementary material for: Age‐specific spectrum of etiological pathogens for viral diarrhea among children in twelve consecutive winter‐spring seasons (2009–2021) in China
Source: J Med Virol. 2022 Apr 26;94(8):3840–6. doi: 10.1002/jmv.27790 (PMC9324210; doi:10.1002/jmv.27790)
Supplement: Supplementary file 1 — Supporting information. [file JMV-94--s001.docx]

**Figure S1. Association between the viral positive rate and the interval between disease onset and hospital admission by logistic regression analysis.**


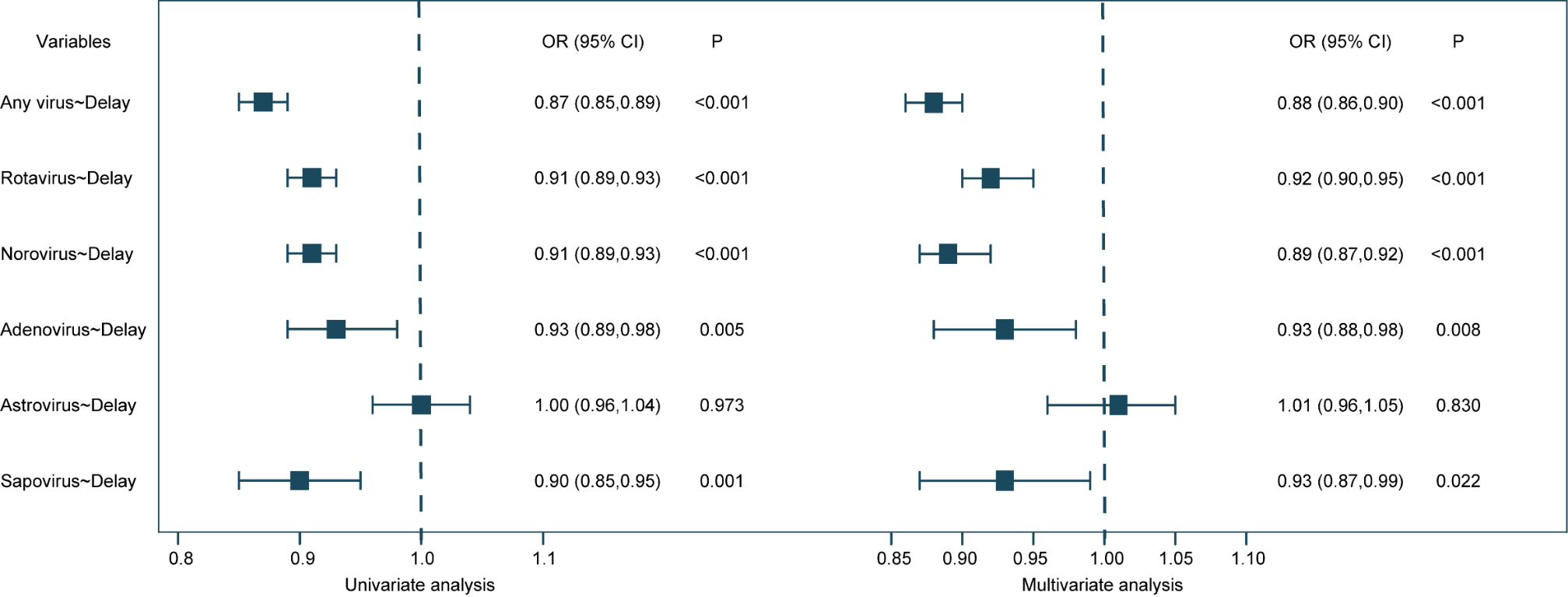


Both results of univariate analysis (Left panel) and the multivariate analysis by adjusting age, sex, month, severity, and residence (Right Panel) were displayed. The box indicates the estimated odds ratio and the interval represents the 95% confidence interval of the odds ratio. *P*-values were corrected using Holm’s method to account for multiple hypothesis testing.

**Figure S2. Enteric virus detection rate and coinfection rate in patients with acute diarrhea.**


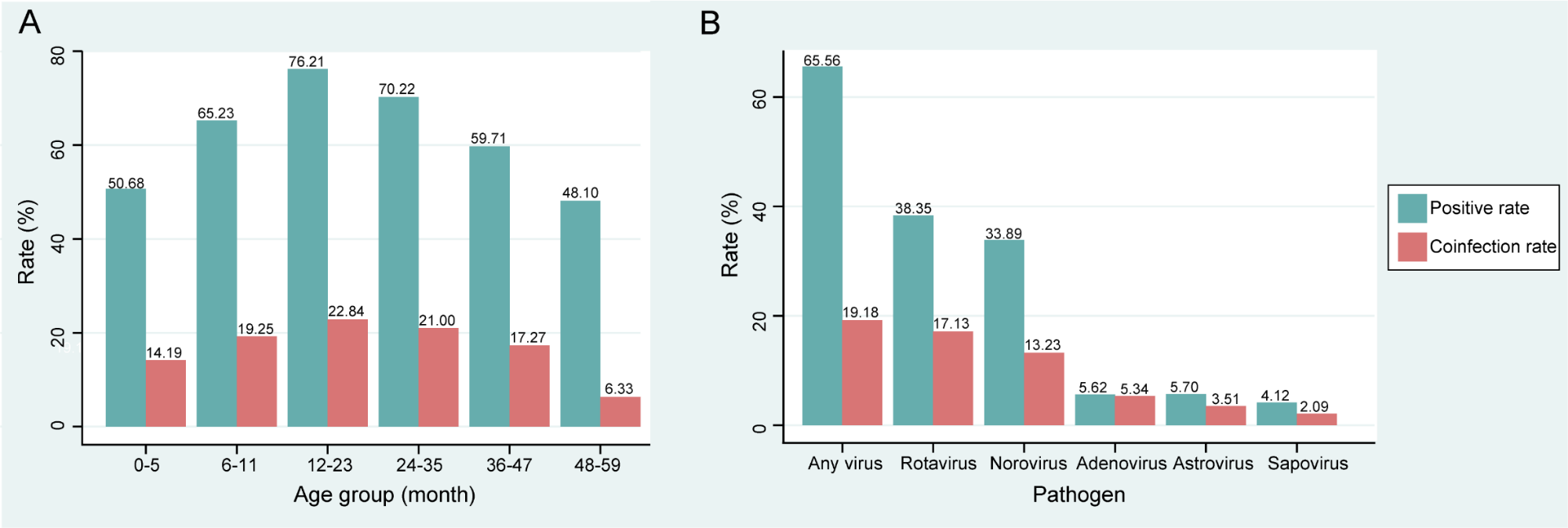


(A) Enteric virus detection rate and coinfection rate in patients with acute diarrhea in different age groups. (B) Enteric virus detection rate and coinfection rate in patients with acute diarrhea for each pathogen. The blue bar indicates the detection rate and the red bar indicates the coinfection rate.

**Table S1. Primers and probes used for detection of enteric viruses.**

| Enteric viruses | Primer and probe | Sequence (5’-3’) | Methods |
| --- | --- | --- | --- |
| Norovirus (GI) | Cog 1F | CGYTGGATGCGITTYCATGA | Real-Time RT-PCR |
|  | Cog 1R | CTTAGACGCCATCATCATTYAC |  |
|  | Probe Ring 1E | FAM-TGGACAGGRGAYCGC-MGBNFQ |  |
| Norovirus (GII) | Cog 2F | CARGARBCNATGTTYAGRTGGATGAG | Real-Time RT-PCR |
|  | Cog 2R | TCGACGCCATCTTCATTCACA |  |
|  | Probe Ring 2 | Cy5/QUASAR670-TGGGAGGGCGATCGCAATCT-BHQ2 |  |
| Adenovirus | Ad1 | TTCCCCATGGCICAYAACAC | PCR |
|  | Ad2 | CCCTGGTAKCCRATRTTGTA |  |
| Astrovirus | Mon269 | CAACTCAGGAAACAGGGTGT | RT-PCR |
|  | Mon270 | TCAGATGCATTGTCATTGGT |  |
| Sapovirus | SLV-5317 | CTCGCCACCTACRAWGCBTGGTT | RT-PCR |
|  | SLV-5749 | CGGRCYTCAAAVSTACCBCCCCA |  |

**Table S2. The pathogen spectrum of enteric viruses in patients with acute diarrhea in Chongqing, China, 2009-2021.**

| **Characteristics** | **Rotavirus (n=1945)** | |  | **Norovirus (n=1719)** | |  | **Astrovirus (n=289)** | |  | **Adenovirus (n=285)** | |  | **Sapovirus (n=209)** | |
| --- | --- | --- | --- | --- | --- | --- | --- | --- | --- | --- | --- | --- | --- | --- |
|  | **Rank** | **Proportion** |  | **Rank** | **Proportion** |  | **Rank** | **Proportion** |  | **Rank** | **Proportion** |  | **Rank** | **Proportion** |
| **All** | 1 | 1945 (43.74) |  | 2 | 1719 (38.66) |  | 3 | 289 (6.50) |  | 4 | 285 (6.41) |  | 5 | 209 (4.70) |
| **Gender** |  |  |  |  |  |  |  |  |  |  |  |  |  |  |
| Boy | 1 | 1126 (41.91) |  | 2 | 1088 (40.49) |  | 4 | 169 (6.29) |  | 3 | 173 (6.44) |  | 5 | 131 (4.88) |
| Girl | 1 | 819 (46.53) |  | 2 | 631 (35.85) |  | 3 | 120 (6.82) |  | 4 | 112 (6.36) |  | 5 | 78 (4.43) |
| **Age group (months)** |  |  |  |  |  |  |  |  |  |  |  |  |  |  |
| 0-5 | 1 | 299 (43.15) |  | 2 | 275 (39.68) |  | 3 | 53 (7.65) |  | 4 | 43 (6.20) |  | 5 | 23 (3.32) |
| 6-11 | 2 | 707 (41.98) |  | 1 | 725 (43.05) |  | 4 | 90 (5.34) |  | 3 | 102 (6.06) |  | 5 | 60 (3.56) |
| 12-23 | 1 | 746 (46.11) |  | 2 | 579 (35.78) |  | 4 | 96 (5.93) |  | 3 | 111 (6.86) |  | 5 | 86 (5.32) |
| 24-35 | 1 | 128 (42.81) |  | 2 | 94 (31.44) |  | 3 | 37 (12.37) |  | 5 | 19 (6.35) |  | 4 | 21 (7.02) |
| 36-47 | 1 | 46 (41.82) |  | 2 | 31 (28.18) |  | 4 | 9 (8.18) |  | 5 | 9 (8.18) |  | 3 | 15 (13.64) |
| 48-59 | 1 | 19 (44.19) |  | 2 | 15 (34.88) |  | 3 | 4 (9.30) |  | 5 | 1 (2.33) |  | 4 | 4 (9.30) |
| **Residence** |  |  |  |  |  |  |  |  |  |  |  |  |  |  |
| Urban | 1 | 1713 (43.59) |  | 2 | 1498 (38.12) |  | 4 | 256 (6.51) |  | 3 | 264 (6.72) |  | 5 | 199 (5.06) |
| Rural | 1 | 222 (44.67) |  | 2 | 212 (42.66) |  | 3 | 32 (6.44) |  | 4 | 21 (4.23) |  | 5 | 10 (2.01) |
| **Severity** |  |  |  |  |  |  |  |  |  |  |  |  |  |  |
| Non-severe | 1 | 1578 (42.66) |  | 2 | 1442 (38.98) |  | 4 | 247 (6.68) |  | 3 | 250 (6.76) |  | 5 | 182 (4.92) |
| Severe | 1 | 367 (49.06) |  | 2 | 277 (37.03) |  | 4 | 38 (5.08) |  | 3 | 39 (5.21) |  | 5 | 27 (3.61) |

Note: Data are n (detection rate%) unless otherwise indicated. The positive proportion of each pathogen was calculated by dividing the number of positive samples by the total number of positive samples. The ranking was ordered based on the proportion.

| **Table S3. Positive rate of enteric viruses by sex, age, residence and severity in patients with acute diarrhea in Chongqing, China, 2009-2021.** | | | | | | | | | | | | | | | | | |
| --- | --- | --- | --- | --- | --- | --- | --- | --- | --- | --- | --- | --- | --- | --- | --- | --- | --- |
| **Enteric viruses** | **All patients**  **(n=5072)** | **Sex** | | ***P***  **value** | **Age group (month)** | | | | | | ***P***  **value** | **Residence** | | ***P* value** | **Severity** | | ***P***  **value** |
|  |  | **Boy**  **(n=****3056)** | **Girl**  **(n=2016)** |  | **0-5**  **(n=1036)** | **6-11**  **(n=1927)** | **12-23**  **(n=1572)** | **24-35**  **(n=319)** | **36-47**  **(n=139)** | **48-59**  **(n=79)** |  | **Urban**  **(n=4483)** | **Rural**  **(n=552)** |  | **Non-sev**  **(n=4342)** | **Severe**  **(n =730)** |  |
| **Any virus** | 3325 (65.56) | 2001 (65.48) | 1324 (65.67) | 0.885 | 525 (50.68) | 1257 (65.23) | 1198 (7.21) | 224 (70.22) | 83 (59.71) | 38 (48.10) | **<0.001** | 2927 (65.29) | 380 (68.84) | 0.097 | 2764 (63.66) | 561 (76.85) | **<0.001** |
| **Rotavirus** | 1945 (38.35) | 1126 (36.85) | 819 (40.63) | **0.007** | 299 (28.86) | 707 (36.69) | 746 (47.46) | 128 (40.13) | 46 (33.09) | 19 (24.05) | **<0.001** | 1713 (38.21) | 222 (40.22) | 0.360 | 1578 (36.34) | 367 (50.27) | **<0.001** |
| **Norovirus** | 1719 (33.89) | 1088 (35.60) | 631 (31.30) | **0.002** | 275 (26.54) | 725 (37.62) | 579 (36.83) | 94 (29.47) | 31 (22.30) | 15 (18.99) | 0.971 | 1498 (33.42) | 212 (38.41) | **0.019** | 1442 (33.21) | 277 (37.95) | **0.012** |
| **Astrovirus** | 289 (5·70) | 169 (5·53) | 120 (5.95) | 0.525 | 53 (5.12) | 90 (4.67) | 96 (6.11) | 37 (11.60) | 9 (6.47) | 4 (5.06) | **0.003** | 256 (5.71) | 32 (5.80) | 0.934 | 250 (5.76) | 39 (5.34) | 0.654 |
| **Adenovirus** | 285 (5.62) | 173 (5.66) | 112 (5.56) | 0.873 | 43 (4.15) | 102 (5.29) | 111 (7.06) | 19 (5.96) | 9 (6.47) | 1 (1.27) | 0.086 | 264 (5.89) | 21 (3.80) | **0.046** | 247 (5.69) | 38 (5.21) | 0.600 |
| **Sapovirus** | 209 (4.12) | 131 (4.29) | 78 (3.87) | 0.464 | 23 (2.22) | 60 (3.11) | 86 (5.47) | 21 (6.58) | 15 (10.79) | 4 (5.06) | **<0.001** | 199 (4.44) | 10 (1.81) | **0.003** | 182 (4.19) | 27 (3.70) | 0.535 |

Note: Data are n (detection rate%) unless otherwise indicated.

| **Table S4. Enteric virus detection rate between boys and girls stratified by age group in patients with acute diarrhea.** | | | | | | | | | | | | | | | | | | |
| --- | --- | --- | --- | --- | --- | --- | --- | --- | --- | --- | --- | --- | --- | --- | --- | --- | --- | --- |
| **Enteric viruses** | **0-5 month (n=1036)** | | ***P* value** | **6-11 month (n=1927)** | | ***P* value** | **12-23 month (n=1572)** | | ***P* value** | **24-35 month (n=319)** | | ***P* value** | **36-47 month (n=139)** | | ***P* value** | **48-59 month (n=139)** | | ***P* value** |
|  | **Boy**  **(n=600)** | **Girl**  **(n=436)** |  | **Boy**  **(n=1172)** | **Girl**  **(n=755)** |  | **Boy**  **(n=955)** | **Girl**  **(n=617)** |  | **Boy**  **(n=191)** | **Girl**  **(n=128)** |  | **Boy**  **(n=91)** | **Girl**  **(n=48)** |  | **Boy**  **(n=47)** | **Girl**  **(n=32)** |  |
| **Any virus** | 303(50.50) | 222(50.92) | 0.894 | 767(65.44) | 490(64.90) | 0.807 | 723(75.71) | 475(76.99) | 0.561 | 130(68.06) | 94(73.44) | 0.304 | 57(62.64) | 26(54.17) | 0.333 | 57(62.64) | 26(54.17) | 0.333 |
| **Rotavirus** | 168(28.00) | 131(30.05) | 0.473 | 409(34.90) | 298(39.47) | **0.042** | 438(45.86) | 308(49.92) | 0.116 | 72(37.70) | 56(43.75) | 0.280\ | 29(31.87) | 17(35.42) | 0.672 | 29(31.87) | 17(35.42) | 0.672 |
| **Norovirus** | 169(28.17) | 106(24.31) | 0.165 | 466(39.76) | 259(34.30) | **0.016** | 367(38.43) | 212(34.36) | 0.102 | 56(29.32) | 38(29.69) | 0.944 | 23(25.27) | 8(16.67) | 0.246 | 23(25.27) | 8(16.67) | 0.246 |
| **Adenovirus** | 22(3.67) | 21(4.82) | 0.360 | 67(5.72) | 35(4.64) | 0.301 | 66(6.91) | 45(7.29) | 0.773 | 12(6.28) | 7(5.47) | 0.763 | 6(6.59) | 3(6.25) | 1.000 | 6(6.59) | 3(6.25) | 1.000 |
| **Astrovirus** | 29(4.83) | 24(5.51) | 0.628 | 57(4.86) | 33(4.37) | 0.617 | 55(5.76) | 41(6.65) | 0.474 | 21(10.99) | 16(12.50) | 0.681 | 5(5.49) | 4(8.33) | 0.776 | 5(5.49) | 4(8.33) | 0.776 |
| **Sapovirus** | 16(2.67) | 7(1.61) | 0.252 | 36(3.07) | 24(3.18) | 0.895 | 54(5.65) | 32(5.19) | 0.690 | 12(6.28) | 9(7.03) | 0.792 | 10(10.99) | 5(10.42) | 0.918 | 10(10.99) | 5(10.42) | 0.918 |

Note: Data are n (detection rate%) unless otherwise indicated.

| **Table S5. Enteric virus detection rate between urban and rural areas stratified by age group in patients with acute diarrhea.** | | | | | | | | | | | | | | | | | | |
| --- | --- | --- | --- | --- | --- | --- | --- | --- | --- | --- | --- | --- | --- | --- | --- | --- | --- | --- |
| **Enteric viruses** | **0-5 month (n=1036)** | | ***P* value** | **6-11 month (n=1927)** | | ***P* value** | **12-23 month (n=1572)** | | ***P* value** | **24-35 month (n=319)** | | ***P* value** | **36-47 month (n=139)** | | ***P* value** | **48-59 month (n=139)** | | ***P* value** |
|  | **Urban**  **(n=903)** | **Rural**  **(n=126)** |  | **Urban**  **(n=1688)** | **Rural**  **(n=225)** |  | **Urban**  **(n=1395)** | **Rural**  **(n=165)** |  | **Urban**  **(n=293)** | **Rural**  **(n=23)** |  | **Urban**  **(n=131)** | **Rural**  **(n=7)** |  | **Urban**  **(n=73)** | **Rural**  **(n=6)** |  |
| **Any virus** | 451(49.94) | 71(56.35) | 0.178 | 1093(64.75) | 156(69.33) | 0.175 | 1066(76.42) | 127(76.97) | 0.874 | 206(70.31) | 16(69.57) | 0.940 | 77(58.78) | 6(85.71） | 0.307 | 34(46.58) | 4(66.67) | 0.602 |
| **Rotavirus** | 260(28.79) | 38(30.16) | 0.752 | 620(36.73) | 83(36.89) | 0.963 | 652(46.74) | 91(55.15) | **0.041** | 119(40.61) | 7(30.43) | 0.337 | 45(34.35) | 1(14.29) | 0.493 | 17(23.29) | 2(33.33) | 0.955 |
| **Norovirus** | 229(25.36) | 44(34.92) | **0.023** | 627(37.14) | 94(41.78) | 0.178 | 519(37.20) | 58(35.15) | 0.606 | 84(28.67) | 9(39.13) | 0.289 | 26(19.85) | 5(71.43) | **0.007** | 13(17.81) | 2(33.33) | 0.696 |
| **Adenovirus** | 39(4.32) | 4(3.17) | 0.548 | 92(5.45) | 10(4.44) | 0.528 | 105(7.53) | 6(3.64) | 0.066 | 19(6.48) | 0 (0) | 0.421 | 8(6.11) | 1(14.29) | 0.383 | 1(1.37) | 0 (0) | 1.000 |
| **Astrovirus** | 47(5.20) | 6(4.76) | 0.833 | 74(4.38) | 15(6.67) | 0.127 | 88(6.31) | 8(4.85) | 0.461 | 34(11.60) | 3(13.04) | 1.000 | 9(6.87) | 0 | 1.000 | 4(5.48) | 0 (0) | 1.000 |
| **Sapovirus** | 23(2.55) | 0 | 0.136 | 56(3.32) | 4(1.78) | 0.213 | 81(5.81) | 5(3.03) | 0.140 | 21(7.17) | 0 (0) | 0.371 | 14(10.69) | 1(14.29) | 0.562 | 4(5.48) | 0 (0) | 1.000 |

Note: Data are n (detection rate%) unless otherwise indicated.

| **Table S6. Enteric virus detection rate between non-severe and severe patients with acute diarrhea in different age groups.** | | | | | | | | | | | | | | | | | | |
| --- | --- | --- | --- | --- | --- | --- | --- | --- | --- | --- | --- | --- | --- | --- | --- | --- | --- | --- |
| **Enteric viruses** | **0-5 month (n=1036)** | | ***P* value** | **6-11 month (n=1927)** | | ***P***  **value** | **12-23 month (n=1572)** | | ***P***  **value** | **24-35 month (n=319)** | | ***P* value** | **36-47 month (n=139)** | | ***P* value** | **48-59 month (n=139)** | | ***P* value** |
|  | **Non-severe**  **(n=933)** | **Severe**  **(n=103)** |  | **Non-severe**  **(n=1653)** | **Severe**  **(n=274)** |  | **Non-severe**  **(n=1294)** | **Severe**  **(n=278)** |  | **Non-severe**  **(n=273)** | **Severe**  **(n=46)** |  | **Non-severe**  **(n=125)** | **Severe**  **(n=14)** |  | **Non-severe**  **(n=64)** | **Severe**  **(n=15)** |  |
| **Any virus** | 467(50.05) | 58(56.31) | 0.228 | 1052(63.64) | 205(74.82) | **<0.001** | 957(73.96) | 241(86.69) | **<0.001** | 189(69.23) | 35(76.09) | 0.347 | 72(57.60) | 11(78.57) | 0.129 | 27(42.19) | 11(73.33) | **0.030** |
| **Rotavirus** | 265(28.40) | 34(33.00) | 0.327 | 586(35.45) | 121(44.16) | **0.006** | 573(44.28) | 173(62.23) | **<0.001** | 104(38.10) | 24(52.17) | 0.072 | 38(30.40) | 8(57.14) | 0.086 | 12(18.75) | 7(46.67) | 0.052 |
| **Norovirus** | 241(25.83) | 34(33.00) | 0.117 | 605(36.60) | 120(43.80) | **0.023** | 475(36.71) | 104(37.41) | 0.826 | 82(30.04) | 12(26.09) | 0.587 | 27(21.60) | 4(28.57) | 0.798 | 12(18.75) | 3(20.00) | 1.000 |
| **Adenovirus** | 38(4.07) | 5(4.85) | 0.907 | 93(5.62) | 9(3.28) | 0.109 | 89(6.88) | 22(7.91) | 0.541 | 19(6.96) | 0 (0) | 0.131 | 7(5.60) | 2(14.29) | 0.225 | 1(1.56) | 0(0) | 1.000 |
| **Astrovirus** | 49(5.25) | 4(3.88) | 0.550 | 81(4.90) | 9(3.28) | 0.240 | 78(6.03) | 18(6.47) | 0.778 | 30(10.99) | 7(15.22) | 0.407 | 9(7.20) | 0 (0) | 0.598 | 3(4.69) | 1(6.67) | 0.577 |
| **Sapovirus** | 23(2.46) | 0 | 0.208 | 49(2.96) | 11(4.01) | 0.354 | 73(5.64) | 13(4.68) | 0.521 | 20(7.33) | 1(2.17) | 0.326 | 14(11.20) | 1(7.14) | 0.992 | 3(4.69) | 1(6.67) | 0.577 |

Note: Data are n (detection rate%) unless otherwise indicated.

**Table S7.** **Enteric virus detection rate in different seasons in pediatric patients with acute diarrhea.**

| **Enteric viruses** | **Spring**  **（n=770）** | **Summer**  **（n=747）** | **Autumn**  **（n=1767）** | **Winter**  **（n=1788）** | ***P* value** |
| --- | --- | --- | --- | --- | --- |
| Any virus | 431 (55.97) | 391 (52.34) | 1246 (70.51) | 1257 (70.30) | <0.001 |
| Rotavirus | 262 (34.03) | 167 (22.36) | 599 (33.90) | 917 (51.29) | <0.001 |
| Norovirus | 200 (25.97) | 248 (33.20) | 800 (45.27) | 471 (26.34) | <0.001 |
| Adenovirus | 64 (8.31) | 62 (8.30) | 72 (4.07) | 87 (4.87) | <0.001 |
| Astrovirus | 39 (5.06) | 49 (6.56) | 90 (5.09) | 111 (6.21) | 0.299 |
| Sapovirus | 19 (2.47) | 28 (3.75) | 98 (5.55) | 64 (3.58) | 0.001 |

Note: Data are n (detection rate%) unless otherwise indicated.
